# Effect of antiplatelet persistence on long-term mortality and predictors of non-persistence in ischemic stroke patients 75 years and older: a nationwide cohort study

**DOI:** 10.1186/s12877-021-02171-4

**Published:** 2021-04-07

**Authors:** Seung Jae Kim, Oh Deog Kwon, Ho Chun Choi, Eung-Joon Lee, BeLong Cho

**Affiliations:** 1 Department of Family Medicine, Seoul St. Mary’s Hospital, College of Medicine, The Catholic University of Korea, Seoul, Republic of Korea; 2 International Healthcare Center, Seoul St. Mary’s Hospital, College of Medicine, The Catholic University of Korea, Seoul, Republic of Korea; 3 Republic of Korea Navy 2nd Fleet Medical Corps, Pyeongtaek-si, Gyeonggi-do Republic of Korea; 4 Department of Family Medicine, Healthcare System Gangnam Center, Seoul National University Hospital, 39th Floor, Gangnam Finance Center, 152 Teheran-ro, Gangnam-gu, Seoul, 06236 Republic of Korea; 5 Department of Neurology, Seoul National University Hospital, 101 Daehak-ro, Jongno-gu, Seoul, 03080 Republic of Korea; 6 Department of Family Medicine, Seoul National University Hospital, Seoul, Republic of Korea

**Keywords:** Ischemic stroke, Antiplatelet, Persistence, Mortality, Patients 75 years and older, Associated factors

## Abstract

**Background:**

We aimed to provide real-world evidence on the benefit of persistence with antiplatelet therapy (APT) on long-term all-cause mortality (ACM) in ischemic stroke patients aged 75 years and older.

**Methods:**

Newly diagnosed ischemic stroke patients aged 75 years and older who initiated aspirin or clopidogrel for the first time were chosen from 2003 to 2010 National Health Insurance Service-National Sample Cohort (NHIS-NSC) of Korea (*n* = 887), a random cohort sample accounting for 2.2% (*n* = 1,017,468) of total population (*n* = 46,605,433). Then subjects were divided into persistent (*n* = 556) and non-persistent (*n* = 321) groups according to the persistent status at 6 months. Survivor analysis was performed between the two groups and predictors of non-persistence were analyzed by multivariate logistic regression analysis. Patients were followed up until death or December 31, 2013.

**Results:**

Non-persistence with APT was significantly associated with increased risk of ACM (adjusted hazard ration [aHR] 2.13, 95% confidence interval [CI] 1.72–2.65), cerebro-cardiovascular disease (CVD) mortality (aHR 2.26, 95% CI 1.57–3.24), and non-CVD mortality (aHR 2.06, 95% CI 1.5702.70). More comorbidities (Charlson comorbidity index score ≥ 6) (adjusted odds ratio [aOR], 2.56, 95% CI 1.43–4.55), older age (aOR 1.52, 95% CI 1.11–2.09 for 80–84 years, aOR 1.73, 95% CI 1.17–2.57 for ≥85 years), and less than 4 total prescribed drugs (aOR 1.54, 95% CI 1.08–2.21) were independent predictors of non-persistence.

**Conclusions:**

Persistent with APT after ischemic stroke featured long-term mortality benefit even in patients aged 75 years and older. Thus, improving APT persistence for ischemic stroke patients in this age group is also recommended by understanding factors associated with non-persistence.

## Background

Stroke is one of the major causes of death in very elderly patients, with nearly one-third of all strokes occurring in patients aged 75 years and older [1–3]. Considering the steady increase in life expectancy, the incidence of stroke is also expected to be more than double over the next 30 years, with a majority of increase in patients aged 75 years and older [3]. Of all strokes, 87% are ischemic [3] and antiplatelet therapy (APT) is the gold standard for secondary prevention [4, 5]. Persistence with APT after ischemic stroke has been proven to result in better clinical outcomes, including decreased vascular death [6, 7]. However, scientific evidence of optimal APT for patients aged 75 years and older have been quite limited since they were substantially excluded from most randomized clinical trials due to age limits or the presence of multiple comorbidities [1, 8, 9]. The benefits of early antiplatelet use after ischemic stroke were similarly observed in patients aged 75 years and older compared to younger patients in a previous meta-analysis [10]. However, it remains unclear whether persistence with APT carries long-term mortality benefits to ischemic stroke survivors in the same old age group. Thus, we analyzed data from a large-scale nationwide claims database in South Korea to gather real-world evidence on the benefits of persistence with APT on long-term mortality in patients aged 75 years and older with ischemic stroke. Furthermore, if persistence with APT were also beneficial to this age group, we aimed to identify the potential predictors of non-persistence to offer a meaningful perspective for improving persistence.

## Methods

### Source of data

We analyzed data from the National Health Insurance Service-National Sample Cohort (NHIS-NSC), a population-based claims database managed by the National Health Insurance Service (NHIS) of South Korea since 2002. The NHIS is the single insurer in South Korea; hence, it offers universal healthcare coverage to all Korean citizens via mandatory enrollment. The NHIS-NSC includes 1,017,468 randomly selected citizens, which makes 2.2% of the total Korean population of 46,605,433. The NHIS-NSC data are collected annually through continuous observations, and are composed of data on qualification (age, sex, household income, health insurance type, residence, death record, etc.), medical service claims (diagnosis record, all insurance-covered healthcare service records, billing statement, etc.), and pharmacy claims (generic name of drugs, prescription dates, total supplied days of medications, dosage and frequency of medication, etc.) [11]. The exemplariness of the NHIS-NSC data including its representativeness and validity are described in previous studies [11, 12].

### Study population

**Fig. 1:**
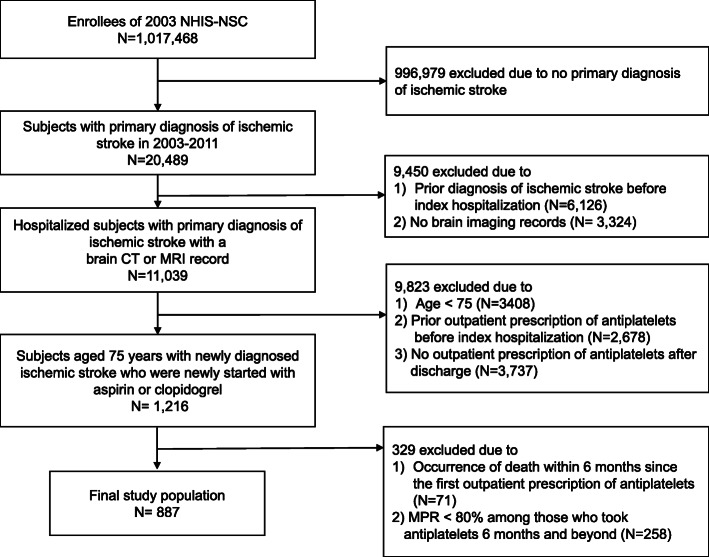
Flow chart of study population selection. NHIS-NSC: National Health Insurance Service-National Sample Cohort; CT: computed tomography; MRI: Magnetic resonance imaging; MPR: medication possession ratio

Newly diagnosed ischemic stroke patients aged 75 years and older who had initiated APT for the first time were selected from the NHIS-NSC between 2003 and 2010. We defined ischemic stroke patients as those who were hospitalized with a primary diagnosis of ischemic stroke (International Classification of Disease, 10th revision, ICD-10: I63, I64, I65, I66) and had a record of brain computed tomography or magnetic resonance imaging during hospitalization. Previous studies that analyzed post-stroke patients from the NHIS-NSC database also adapted this definition [13, 14]. We were able to properly extract newly diagnosed ischemic stroke patients who were newly prescribed with antiplatelets by excluding individuals with prior histories of ischemic stroke diagnosis (I63-I66) or outpatient prescription of antiplatelet agents before the index hospitalization dates. The type of antiplatelet agents was limited to aspirin or clopidogrel, which are the most frequently prescribed antiplatelets for the secondary prevention of ischemic stroke [5, 15, 16], in accordance with the Anatomical Therapeutic Chemical classification code [17]. Other antiplatelet agents besides aspirin or clopidogrel were not included because these were very rarely prescribed during the study period, owing to the fact that they were not covered by the NHIS at that time. Participants without an outpatient prescription of aspirin or clopidogrel after discharge were also excluded. To investigate the impact of non-persistence with APT on long-term mortality, survival analysis was planned according to the status of persistence with APT 6 months after the first prescription. Thus, individuals who died prior to 6 months after the first prescription of antiplatelets were excluded. The non-persistent group was defined as the patients who discontinued antiplatelet intake within 6 months. For the operational definition of the persistent group, adherence to antiplatelets of continuers was obtained using the medication possession ratio (MPR), which was calculated by dividing the total number of days supplied by the number of days in the time interval between the first and the last prescription [18]. We then defined the persistent group as the patients who continued antiplatelet intake for 6 months and beyond with good medication adherence (MPR ≥80%). The 80% of MPR has been established as a reliable threshold for determining good adherence [18, 19] and thus, it is commonly applied when defining persistence with claims data [20, 21]. In effect, a total of 887 patients were selected for the final study population (Fig. 1).

### Definition of non-persistence with antiplatelets

The outpatient prescription of antiplatelets of every participant were observed until December 31st of the second calendar year from the date of initial outpatient prescription. As a result, prescription of antiplatelets of each participant was observed for at least 2 years. Non-persistence was defined as when the prescription of antiplatelets was discontinued within 6 months without re-initiation for the rest of the observation period. If the participants continued to receive either aspirin or clopidogrel prescriptions during the observation, they were considered as continuers, without regard for drug switching between the two drugs. Patients who were on aspirin and clopidogrel at the same time were viewed as discontinuers only when both agents were discontinued without re-initiation for the remainder of the observation period.

### Outcomes assessment

The primary end point of the present study was all-cause mortality (ACM), whereas cerebro-cardiovascular disease (CVD) mortality and non-CVD mortality were secondary end points. We defined CVD mortality as death events due to cerebrovascular disease (ischemic and hemorrhagic stroke, I60–69) and ischemic heart disease (I20–25). All other death events that were not associated with CVD mortality were regarded as non-CVD mortality. The dates and causes of death were derived from the death records, which are part of the qualification data of the NHIS-NSC. Each patient was followed up from the date of first outpatient antiplatelet prescription to the date of death or December 31, 2013, whichever came first.

### Statistical analysis

We calculated the crude and adjusted hazard ratios (aHRs) with a 95% confidential intervals (CIs) for all-cause death, CVD death, and non-CVD death between the APT persistent and non-persistent groups by using the Cox proportional hazard regression model. The Kaplan-Meier method was applied to construct the survival curves. The proportional hazards assumption in the Cox regression was examined using Schoenfeld residuals method and it was all satisfied. Univariate and multivariate logistic regression analyses were also conducted between the two groups to investigate the predictors of non-persistence with APT. Both the Cox proportional hazards model and the multivariate analysis for predictors of non-persistence were adjusted for age, sex, household income, residential area, health insurance type, Charlson comorbidity index (CCI), and number of prescribed drugs. The CCI score was assessed at the time of first outpatient prescription of antiplatelets for each patient, based on ICD-10 codes [22] while the number of prescribed drugs was calculated by averaging the total number of prescribed medications per each outpatient visits during the observation period of antiplatelet prescriptions. All data collection and statistical analyses were carried out with STATA version 14.1 (Stata Corp., College Station, TX, USA). *P*-values < 0.05 were considered to be statistically significant.

## Results

### Baseline characteristics of study population

The baseline characteristics of the total study population, patients who discontinued APT prematurely within 6 months and those who continued APT for 6 months and beyond are presented in Table 1. Among the 887 total study population, 36.2% (*N* = 321) were in the non-persistent group and 63.8% (*N* = 566) were in the persistent group. Of the total patients, 42.1% were male and 57.9% were female. The mean age of all the patients was 80.5 ± 3.7 years. Specifically, 55.1% were in age 75–79 years group, 29.6% were in age 80–84 years group, and the remaining 15.3% were aged 85 years and older. The majority of the individuals (81.4%) resided in urban areas, and 71.2% were identified to belong to the upper-middle income class. Nearly all of the patients (90.8%) were receiving insurance coverage through Medicare, while 9.2% benefited from Medical Aid. Regarding comorbidity status, those with CCI scores less than 3 accounted for 47.3% of the total study population, 46.8% had CCI scores of 3–5, and 5.9% had CCI scores of 6 and higher. In terms of the polypharmacy status, 17.4% of patients were prescribed a total of 4 drugs, 63.8% had 4 to 7 in total, and 18.8% were prescribed with a total of at least 8 drugs. The mean duration of APT of total patients, persistent, and non-persistent group was 465.8 ± 372.5 days, 703 ± 245.5 days, and 47 ± 55.4 days, respectively. The non-persistent group was slightly more likely to be older, with more income and comorbidities, but with a lesser total number of prescribed drugs compared to the persistent group. Though the differences were all insignificant, this group also had a slightly higher percentage of females, rural residents, and Medical Aid beneficiaries compared to the persistent group.

**Table 1:** Baseline characteristics of study population

Characteristics	All n (%) or mean ± SD	Non-persistent (< 6 months) n (%) or mean ± SD	Persistent (≥ 6 months) n (%) or mean ± SD	*p* value
Total	887 (100%)	321 (100%)	566 (100%)	
Sex				0.889
Male	373 (42.1%)	134 (41.7%)	239 (42.2%)	
Female	514 (57.9%)	187 (58.3%)	327 (57.8%)	
Age (years)	80.5 ± 3.7	81.0 ± 3.8	80.2 ± 3.6	0.007
75-79	489 (55.1%)	155 (48.3%)	334 (59.0%)	
80-84	262 (29.6%)	106 (33.0%)	156 (27.6%)	
≥ 85	136 (15.3%)	60 (18.7%)	76 (13.4%)	
Household income				0.046
Low	256 (28.8%)	135 (42.0%)	287 (50.7%)	
Middle	209 (23.6%)	83 (25.9%)	126 (22.3%)	
High	422 (47.6%)	103 (32.1%)	153 (27.0%)	
Residential area				0.191
Urban	722 (81.4%)	254 (79.1%)	468 (82.7%)	
Rural	165 (18.6%)	67 (20.9%)	98 (17.3%)	
Type of health insurance				0.423
Medicare	805 (90.8%)	288 (89.7%)	517 (91.3%)	
Medical aid	82 (9.2%)	33 (10.3%)	49 (8.7%)	
Charlson comorbidity index				0.009
1-2	420 (47.3%)	143 (44.6%)	277 (48.9%)	
3-5	415 (46.8%)	149 (46.4%)	266 (47.0%)	
≥ 6	52 (5.9%)	29 (9.0%)	23 (4.1%)	
Number of prescribed medications				0.009
< 4	154 (17.4%)	68 (21.2%)	86 (15.2%)	
4-7	566 (63.8%)	184 (57.3%)	382 (67.5%)	
≥ 8	167 (18.8%)	69 (21.5%)	98 (17.3%)	
Mean duration of antiplatelets (days)	465.8 ± 372.5	47.8 ± 55.4	703.0 ± 245.4	0.000

*Abbreviations SD* Standard deviation

### The impact of persistence with APT on long-term mortality

**Fig. 2:**
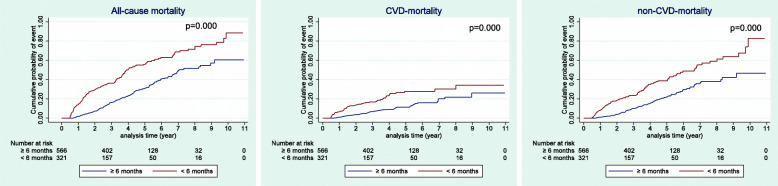
Kaplan-Meier curves of cumulative probability of event after ischemic stroke between the antiplatelet continuers and discontinuers. CVD: cerebro-cardiovascular disease; non-CVD: non-cerebro-cardiovascular disease

The association between persistence with APT and long-term mortality (ACM, CVD mortality, and non-CVD mortality) is shown in Table 2. During the follow-up period (median follow-up: 3.17 years, and maximum follow-up: 11 years), there were 173 total cases of all-cause death (63 CVD deaths, and 110 non-CVD deaths) in the non-persistent group and 168 cases in the persistent group (59 CVD deaths and 109 non-CVD deaths). Early discontinuation of APT within 6 months was significantly associated with an increased risk of ACM (aHR 2.13, 95% CI 1.72–2.65], CVD mortality (aHR 2.26, 95% CI 1.57–3.24), and non-CVD mortality (aHR 2.06 95% CI (1.57–2.70). The Kaplan-Meier survival curves also displayed a significant correlation between persistence with APT and long-term mortality. The difference rates of all-cause death, CVD death, and non-CVD death between the persistent and non-persistent groups remained in a similar range throughout the follow-up period (Fig. 2).

**Table 2:** Association of persistence with antiplatelets on long-term mortality^a^ after ischemic stroke

Duration of antiplatelets	All-cause mortality					CVD mortality					Non-CVD mortality				
	Event No	Crude HR (95% CI)	*p* value	Adjusted HR^b^ (95% CI)	*p* value	Event No	Crude HR (95% CI)	*p* value	Adjusted HR^b^ (95% CI)	*p* value	Event No	Crude HR (95% CI)	*p* value	Adjusted HR^b^ (95% CI)	*p* value
Non-persistent (< 6 months) (*n* = 321)	173	2.35 (1.90-2.91)	0.000	2.13 (1.72-2.65)	0.000	63	2.40 (1.68-3.42)	0.000	2.26 (1.57-3.24)	0.000	110	2.33 (1.78-3.03)	0.000	2.06 (1.57-2.70)	0.000
Persistent (≥ 6 months) (*n* = 556)	168	Reference		Reference		59	Reference		Reference		109	Reference		Reference	

Analysis was performed using Cox proportional hazard regression model

*Abbreviations*: *CVD* Cerebro-cardiovascular disease, *No* Number, *HR* Hazard ratio

^a^Median follow-up was 3.17 years

^b^Adjusted for sex, age, income, residential area, type of insurance, Charlson comorbidity index, and number of prescribed medications

### Predictors of non-persistence with APT

The results of the univariate and multivariate logistic regression analyses between the APT persistent and non-persistent groups are presented in Table 3. Overall, the results of multivariate analysis demonstrated that having more comorbidities (CCI score of 6 and higher) was the most significant factor that predicted the premature discontinuation of APT within 6 months (adjusted odds ratio [aOR] 2.56, 95% confidence interval [CI] 1.08–2.21). Older age (aOR 1.52, 95% CI 1.11–2.09 for age 80–84 years group, aOR 1.73, 95% CI 1.17–2.57 for age ≥ 85 years group) and having less than 4 total prescribed drugs (aOR 1.54, 95% CI 1.08–2.21) were also identified as significant predictors of early discontinuation of APT within 6 months. However, early discontinuation of APT had no significant correlation with other factors, including sex, household income, residential area, and type of insurance.

**Table 3:** Predictors of the early discontinuation of antiplatelets within 6 months after ischemic stroke compared to continuers

Factors	Univariate logistic regression analysis		Multivariate logistic regression analysis	
	Crude OR (95% CI)	*p* value	Adjusted OR^a^(95% CI)	*p* value
Sex				
Male	1 (reference)		1 (reference)	
Female	1.02 (0.77-1.35)	0.889	0.97 (0.73-1.29)	0.857
Age				
75-79	1 (reference)		1 (reference)	
80-84	1.46 (1.07-2.00)	0.016	1.52 (1.11-2.09)	0.009
≥ 85	1.70 (1.15-2.51)	0.007	1.73 (1.17-2.57)	0.007
Income				
High	1 (reference)		1 (reference)	
Middle	1.40 (0.99-1.98)	0.055	1.43 (1.00-2.03)	0.048
Low	1.43 (1.04-1.98)	0.030	1.38 (0.95-2.02)	0.093
Residential area				
Urban	1 (reference)		1 (reference)	
Rural	1.26 (0.89-1.78)	0.191	1.23 (0.86-1.76)	0.256
Health insurance				
Medicare	1 (reference)		1 (reference)	
Medical aid	1.21 (0.76-1.92)	0.423	1.09 (0.63-1.88)	0.758
Charlson comorbidity index				
< 6	1 (reference)		1 (reference)	
≥ 6	2.34 (1.33-4.13)	0.003	2.56 (1.43-4.55)	0.001
Number of prescribed medications				
≥ 4	1 (reference)		1 (reference)	
< 4	1.50 (1.05-2.13)	0.024	1.54 (1.08-2.21)	0.000

^a^Adjusted for sex, age, income, residential area, type of health insurance, Charlson comorbidity index, and number of prescribed medications

## Discussion

In this nationwide retrospective cohort study, we confirmed that even among those aged 75 years and older, persistent APT after initial ischemic stroke was associated with reduced risks for both CVD and non-CVD death. In fact, the risk of mortality of any cause, CVD-related, and non-CVD deaths for those who discontinued APT within 6 months were more than doubled compared to patients who continued APT for 6 months and beyond. The higher risk of CVD mortality in the non-persistent group is presumably due to a higher incidence of recurrent stroke or coronary artery diseases including acute coronary syndromes in this group than in the persistent group. The risk of recurrence after initial ischemic stroke is known to remain elevated for several years [23] and its rates are even higher in elderly patients. The incidence of first-ever [24] ischemic stroke was also an independent risk factor for major cardiovascular events, including incident coronary artery disease, acute coronary syndrome and myocardial infarction in Canadian patients aged 66 years and older [25]. The risk of these events increases even more when APT is discontinued prematurely [6, 7], and this likely resulted in a higher risk of CVD mortality in patients aged 75 years and older in this study. Furthermore, even if patients survive recurrent stroke and acute coronary syndrome, the consequences of these events may lead to complications that increase the risk of non-CVD death. Cognitive and physical dysfunction due to the sequelae of stroke recurrence or acute coronary syndrome may force patients to be hospitalized longer and be more bed-ridden, which would eventually cause more non-CVD deaths due to events such as falls, pulmonary thromboembolism, aspiration pneumonia, various hospital-acquired infections, and depression [26]. This may also further explain our findings. In addition, our Kaplan-Meier curves for cumulative probability of event between the two groups showed that the degree of difference for all-cause death, CVD, and non-CVD death all remained in a similar range throughout the study period. This suggested that APT for at least 6 months after ischemic stroke continued to bring mortality benefits to patients aged 75 years and older over time. Thus, persistence and adherence to antiplatelets after ischemic stroke should also be emphasized to patients in this age group.

When it comes to the factors influencing the non-persistence with APT after ischemic stroke for patients 75 years and older, older age, less total prescribed drugs (< 4 total drugs), and having more comorbidities (CCI score ≥ 6) were positive predictors of premature discontinuation of APT within 6 months.

In terms of age, both the cognitive and physical functions of every individual undoubtedly decline with increasing age, and impairment in these functions have been well-known risk factors for medication non-adherence in the elderly population [27–29]. This trend of older age and lesser medication adherence was consistent with a previous Chinese study that was also conducted among ischemic stroke patients [30].

Regarding polypharmacy, the present study confirmed that taking less than 4 total prescribed drugs was predictive of early discontinuation of APT. This result was quite contrary to the general belief that the more drugs patients have to take, the poorer is their medication adherence. This tendency might be explained by the “health belief model.” [31, 32] According to the health belief model, those who believe they are at lower risks of developing certain diseases are more likely to engage in relatively unhealthy behaviors [31, 32]. Since the general medical condition of patients with 1–3 total prescribed drugs is likely to be better than those with 4 or more total medications, the significance of continuing APT after ischemic stroke may have been less important for them. Other studies that reported a positive relationship between a lower total number of drugs and medication adherence of antihypertensives or statins also used health belief model for the interpretation of their results [33, 34].

Nevertheless, our findings revealed that the health belief model becomes ineffective when the individuals’ general health status was too serious, as having more comorbidities (CCI score ≥ 6) was a notable predictor of non-persistence with antiplatelets after ischemic stroke for patients aged 75 years and older. Various studies have reported that more comorbidities for geriatric ischemic stroke patients are significantly associated with more functional dysfunctions, subsequently limiting activities of daily living [35–38]. We believe that this is the reason for the increased risk of early APT discontinuation in patients with CCI scores of 6 and higher. Having more comorbidities was also confirmed as a risk factor for poorer antihypertensive adherence in a previous study [39].

Interestingly, the conventional socioeconomic risk factors for medication non-adherence, such as household income, type of insurance, and residential area [40–42] were not predictive of APT non-persistence after ischemic stroke in the patients aged 75 years and older. This may imply that for older patients with ischemic stroke, medication adherence is influenced more by an individual’s health condition or therapy-related factors rather than by socioeconomic factors. A Canadian registry-based stroke study also reported that the socioeconomic status of elderly ischemic stroke patients was not a decisive factor in determining better adherence to secondary preventive drugs [43]. Further studies regarding the specific characteristics of factors affecting APT adherence in very elderly ischemic stroke survivors should be performed to confirm these results.

The present study had some meaningful implications. First, by using nationally representative large-scale claims data, we provided real-world evidence that being persistent with APT also had long-term mortality benefits for ischemic stroke patients aged 75 years and older. Although previous studies have confirmed the benefit of early antiplatelet use for geriatric ischemic stroke patients [4, 10], evidence concerning its persistent use in very old ischemic stroke patients has been relatively limited. Second, we included various potential factors, including age, sex, household income, residence, health insurance type, comorbidities, and polypharmacy, that could influence APT persistence as covariates and discovered the independent predictors of non-persistence with APT after ischemic stroke in very old patients.

However, this study also had some limitations. First, the severity of stroke or neurological status could not be assessed due to the nature of the claims database. Second, the particular reasons for early APT discontinuation, such as the possible side effects of APT (bleeding and gastrointestinal toxicity) and the patients’ social history, including smoking and alcohol consumption status, were not addressed because of the lack of information in the NHIS-NSC. Nevertheless, we believe that these missed factors were likely to be distributed evenly to both case and control groups, considering the large sample size and the well-established representativeness of the NHIS-NSC. Third, since our investigation of medication persistence was only limited to APT, patients who switched from APT to anticoagulation therapy, due to the detection of paroxysmal atrial fibrillation (pAF), may have been misplaced into the non-persistent group. However, both the extent and range of work-up for detection of pAF among cryptogenic stroke cases substantially varies from one institution to another, and the majority of institutions are reported not to perform routine electrocardiogram (ECG) at follow-up outpatient visits after discharge [44]. In addition, detection rates of pAF for cryptogenic stroke patients were low (1.4% at 6 months and 2.0% at 12 months) with conventional ECG monitoring, and they were still relatively lower, even with an insertable cardiac monitor with only 8.9% at 6 months and 12.4% at 12 months [45]. Furthermore, even if we did discover all the patients who were detected with pAF, since anticoagulation was performed with only warfarin during our study period, it is technically impossible to properly evaluate the persistence and adherence of warfarin since its dose and prescriptions are adjusted in accordance with patients’ time in the therapeutic range. In addition, the non-persistent group already had significantly higher mortality risk despite the possibility of including those who switched to anticoagulation therapy in the non-persistent group. Thus, the effect of this limitation on the overall results of the present study is less significant. Fourth, since the follow-up of participants of this study was initiated at the time of the first outpatient antiplatelet prescription after discharge from the hospital, there is a possibility of potential immortal time bias. However, considering the fact that majority of ischemic stroke patients in Korea are prescribed with antiplatelets at discharge [46], the time period between discharge and the first outpatient prescription cannot just be regarded as untreated period. Thus, we believe that the impact of potential immortal time bias on the overall results of our study would be relatively small. Lastly, the over-the-counter (OTC) aspirin intake was not reflected because the NHIS-NSC contains only insurance-covered prescription information. However, we supposed that most of the patients would have taken aspirin through prescription rather than OTC purchase, since patients can receive the former at a discounted price via Korean health insurance coverage [47]. In addition, a previous study proved that prescription claims data can provide valid estimates regardless of missed OTC exposures [48].

## Conclusions

In this retrospective population-based study, persistence with APT was markedly associated with a reduced risk for ACM, CVD mortality, and non-CVD mortality in ischemic stroke patients aged 75 years and older. Moreover, older age, having less than 4 total prescribed drugs, and a CCI score of 6 and higher were independent predictors of APT non-persistence. Therefore, improving APT persistence for ischemic stroke patients in this age group is also recommended by understanding factors associated with non-persistence.

## Data Availability

Data used in this study are derived from the National Health Insurance service (NHIS). Data cannot be shared publicly because Korean legal restrictions prohibit the authors from making the data publicly available. Data can only be accessed through the NHIS’ National Health Insurance Data Sharing Service website http://nhiss.nhis.or.kr/bd/ab/bdaba021eng.do . To gain access to NHIS-NSC data, a completed application form, a research proposal and the applicant’s institutional review board (IRB) approval document should be submitted to and reviewed by the Review Committee of Research Support in NHIS. After granting approval, data becomes accessible to an applicant through above mentioned website for a fee.

## Abbreviations

ACM: All-cause mortality
aHR: Adjusted hazard ratio
aOR: Adjusted odds ratio
APT: Antiplatelet therapy
CCI: Charlson comorbidity index
CI: Confidence interval
CVD: Cerebro-cardiovascular disease
ECG: Electrocardiogram
ICD-10: International Classification of Disease, 10th revision
MPR: Medication possession ratio
NHIS-NSC: National Health Insurance Service-National Sample Cohort
OTC: Over-the-counter
pAF: Paroxysmal atrial fibrillation
